# Advances in Variable Rate Technology Application in Potato in The Netherlands

**DOI:** 10.1007/s11540-018-9357-4

**Published:** 2018-02-19

**Authors:** Corné Kempenaar, Thomas Been, Johan Booij, Frits van Evert, Jean-Marie Michielsen, Corné Kocks

**Affiliations:** 1 Department of Agro Systems Research, Wageningen University & Research, PO Box 16, NL, 6700 AA Wageningen, the Netherlands; 2 Department of Toegepast Praktijkonderzoek, Aeres University of Applied Sciences, De Drieslag 4, NL, 8251 JZ Dronten, the Netherlands

**Keywords:** Cost-benefit analysis, Decision support, Information technology, Precision agriculture, Smart farming

## Abstract

Precision agriculture is a farming management concept based on observing, measuring and responding to inter- and intra-field variability in crops. In this paper, we focus on responding to intra-field variability in potato crops and analyse variable rate applications (VRAs). We made an overview of potential VRAs in potato crop management in The Netherlands. We identified 13 potential VRAs in potato, ranging from soil tillage to planting to crop care to selective harvest. We ranked them on availability of ‘proof of concept’ and on-farm test results. For five VRAs, we found test results allowing to make a cost-benefit assessment. These five VRAs were as follows: planting, soil herbicide weed control, N side dress, late blight control and haulm killing. They use one of two types of spatial data: soil maps or biomass index maps. Data on costs and savings of the VRAs showed that the investments in VRAs will pay off under practical conditions in The Netherlands. Savings on pesticide use and N-fertilizer use with the VRAs were on average about 25%, which benefits the environment too. We foresee a slow but gradual adoption of VRAs in potato production. More VRAs will become available given ongoing R&D. The perspectives of VRAs in potatoes are discussed.

## Introduction

Precision agriculture or precision farming (PF) can be defined as doing the right thing, in the right place, at the right time, in the right way (Blackmore et al. 2005). It is a farming management concept based on observing, measuring and responding to inter- and intra-field variability in crops. The potential of PF was first recognised by scientists at the end of the last century (e.g. Bouma 1996), who showed that information on spatial variation of soil properties could be used to optimize nutrient use in crops site-specifically. In the next years, several precision agriculture ‘enabling technologies’ became ready-to-use in farming, such as soil and crop sensors, farm management information systems (FMIS), global navigation satellite systems (GNSS), geographical information systems (GIS) and other information technology (IT) tools, triggering the interest of farmers and agri-food chain partners in PF as well. Farmers and chain partners expect that management processes can be optimized (both economically and ecologically) with PF. New PF services and products are being marketed by companies. Society expects more sustainable production from PF. So, expectations of PF are high (Haverkort and Kempenaar 2016). However, still more research and development is needed before broad adoption of PF can take place, and its expected benefits can be harvested (Kempenaar and Lokhorst 2016).

Precision agriculture technology (PAT) can be categorised as recording, reacting and guidance techniques. In this study, we focus on reacting PATs: variable rate application (VRA). A first objective of this paper is to give an overview on VRA PATs in potato in The Netherlands. VRA is a PF application that uses data on spatial and temporal variation of specific parameters of soil or crop, in order to optimize a crop management activity. Inputs are optimized according to site-specific needs. In each crop, at least 5 and up to 20 VRAs can be identified. Each VRA requires the following: (1) data on spatial and temporal variation of key parameters, (2) a model that translates data into a task map, and (3) an implement that can apply the tasks site-specifically. This all has to be embedded in a supportive IT environment that allows easy and safe use of data, models and advice (task maps in case of VRA).

In this paper, we provide an overview of emerging PA VRAs in potatoes. We identified five (near) mature VRAs based on on-farm tests in The Netherlands during the last 5–10 years. The second objective is to analyse the on-farm test results in order to determine if there is a business case for VRAs in potatoes. We did a cost-benefit assessment (CBA) on the five emerging PA VRAs. The results are discussed in the final section.

## Materials and Methods

We carried out a desk study in 2017 to identify possible VRAs in potatoes, to summarize on-farm experimental data with mature VRAs and to do a cost-benefit analysis (CBA).

We started with describing the current situation of potato production in The Netherlands. We decided to focus on production of French fries potatoes on clay soil, for which we had available quantitative information on costs and benefits of the cropping system for reference (see Annex Table 2). We then identified potential and demonstrated existing VRAs in potato production based on the table in the Annex, additional literature search and expert judgement. We rated the VRAs’ readiness-to-use in potato production. The rating categories were as follows:

- 1 = potential of the VRA has been identified, but no proof of concept or uses known;
- 2 = potential identified plus proof of concept available;
- 3 = potential identified, proof of concept available plus on-farm test results available.

For each VRA, we summarized the state of the art of the VRA and described any known data on costs and benefits of it. We identified 5 VRAs for which data were available to allow a first cost-benefit assessment with on-farm experimental data.

We then carried out a CBA for individual and combined VRAs, assuming some of the PAT can be used in different VRAs. Finally, we discuss the results of the desk study.

## Results and Discussion

In this section, we first present a summary of VRAs in potatoes. Secondly, we define baseline data for the CBA. Details on individuals VRA are given in the next sections. Finally, we discuss the results of the CBA.

### Potato and Precision Agriculture

Potato is the most important arable crop in Dutch farming. It is grown on ca. 20% of the arable land, with a total production of 7.5 million Mg year^−1^ and a farm-level value of € 800 million (Haverkort et al. 2008). The crop has received much attention from precision agriculture scientists and research and development specialists in The Netherlands. We identified at least 13 possible precision agriculture applications (PAAs)/VRAs in potatoes (Table 1). For five of these, we found experimental results that can be used in a CBA of precision agriculture (PA) in potatoes. These five PAAs are VRA planting, weed control, N side dress, late blight control, and potato haulm killing.

**Table 1:** Overview of different variable rate crop management operations in potato crops

Variable rate application type	# of operations per crop	Timing of operations	Concept	Status^1^
Soil tillage	1–3	Autumn and spring		1
Fertilizer application (base)	1–3	Before or at planting	Use of NIR sensors to optimize animal manure dose rate, and as applied maps	2
Soil pathogen control	1	Before or at planting	Spot application of nematicides based on maps of nematode infestations	2,3
Planting	1	At planting	VRA planting density based on soil and shadow maps	2,3
Ridging	1	At or after planting		1
Weed control, herbicides	1–3	Early in growth season	VRA soil herbicides based on soil map, and/or spot application	2,3
Fertilizer application (N top dress)	1–2	During growth season	VRA N based on biomass map and DSS	2,3
Disease control, fungicides	5–15	During growth season	VRA fungicides based on biomass map and DSS	2,3
Insect control, insecticides	1–3			1
Virus control	2–4	During growth season	Detection of virus-infected plants	2
Irrigation	0–3	During growth season	Timing based on soil water balance and DSS	2
Potato haulm killing	1–2	Before harvest	VRA N based on biomass map and DSS	2,3
Selective harvest				1

1 potential has been identified, but no proof of concept or uses known, 2 potential plus proof of concept, 3 potential plus proof of concept and on farm test results

### Baseline Data

In the CBA, we focus on a region in The Netherlands where processing potatoes are widely grown. This region is Flevoland with young marine clay soil. We used standard KWIN data on agricultural budgeting and practices in Dutch farming (PPO 2015) to list quantities and values of inputs and outputs in potato production (Table 2 in Annex). KWIN lists inputs and outputs needed for 1 ha of crop (in this case potato) but it excludes the cost of the farmer’s own labour, the cost of using the farm’s own machines and the cost of the farm’s capital. The value of these items cannot be ascertained straightforwardly as it is highly dependent on the specific farm. We assumed that the value of the farmer’s labour and the farm’s machines is one-third of the value of the potatoes produced (PAGV 1995).

#### Soil Tillage

Fuel use while tilling the soil and penetrometer data have been identified as ways to map soil compaction of agricultural fields and to identify zones in those fields that require extra tillage (e.g. van Dijk et al. 2016). However, we did not find publications on science-based models for variable rate soil tillage nor did we find quantitative test results of this type of VRA. For this reason, we did not address this potential PAA in the PA CBA.

#### Fertilizer Application 1 (Base)

Although variable rate nutrient application is considered to be one of the most promising PAAs (Bouma 1996), the number of variable rate (VR) fertilizer applications in arable farming practice is still limited. In the CBA, we did not address VR base application of fertilizers in potato because we did not find science-based concepts nor quantitative data. Further on, we address VR N top-dress application in potato. Another promising PAT we mention in this section is the use of near-infra-red (NIR) sensors to measure the N, P, K and other contents in liquid animal manure (slurry), allowing more homogeneous application of the nutrients on agricultural fields (Hoving et al. 2015). As applied maps, this PAT can also be used to check and identify unfertilized or double-treated zones within agricultural fields. This PAT for optimization of liquid manure use is not considered in the PA CBA.

#### Soil- and Seed-Borne Pathogens (Fungi, Nematodes)

Soil-borne diseases occur in patches. Been and Dantuma (2013) published results of an online task map application for site-specific granulate application to control nematodes, using soil analysis data and the NemaDecide decision support system (Been et al. 2007). This PAT as well as methods to combat seed-borne pathogens were not studied in the PA CBA.

#### Planting and Ridging

Mechanical planting of potatoes in rows is the region of our CBA. The optimal planting depth of tubers and the spacing between tubers in the rows may differ depending on conditions (soil, climate, variety) and objectives (production of seed, processing or ware potatoes) (Allen 1978). Modern planters can vary spacing between potatoes in the row. This PAT allows the adjustment of tuber density to soil and environmental conditions. Malda and Specken (2011) published a first study on variable rate seeding of potatoes based on soil maps. In the EU IoF2020 project, we studied VR seeding of potatoes based on lutum content of the soil, distance to tram line of the sprayer and yes or no shading by obstacles like trees. Where lutum content of the field was relatively low and/or shading by trees was expected, seed spacing was larger and vice versa (high lutum content and no shading, smaller spacing). Next to tram lines, seed spacing was smaller. Preliminary results showed up to 4% more yield, and a more homogeneous produce. In the CBA, we assumed 2% more yield. Savings on input of seeds were not accounted for because the farmers involved aimed at better distribution of the seeds rather than reducing seed input. Soil maps were obtained with a VERIS MSP3 or EM38 soil sensor system. Cost of the map is ca 150 € ha^−1^. The map can be used 3 to 5 years in a row and may have at least two uses (in VRA planting and soil herbicides). We assumed an annual cost of 25 € ha^−1^ in the CBA. Additional machine costs for this PAT are ca 30 € ha^−1^ year^−1^ (derived from € 15,000 extra per machine, depreciation in 5 years and 100 ha per machine per year).

We did not find publications on variable ridging in potatoes, and this was not considered in the PA CBA.

#### Weed Control

Site-specific weed control is another promise of PA and a way to reduce herbicide use in crops (e.g. Christensen et al. 2009). In this paper, we addressed VRA of soil herbicides in potatoes using soil maps. We did not address PAT for weed detection and VR post-emergence weed control with contact herbicides or with non-chemical control, because this is not yet practical or not applied in potato crops.

The amount of soil herbicides used in crops can be reduced by adjusting the dosage to the local soil condition. In particular, soil herbicides are more effective in zones where soil organic matter and/or lutum content are low. The application rate of soil herbicides can be lowered in those zones without affecting their efficacy. Results on VRA of soil herbicides and a VRA model have been published (Kempenaar et al. 2014a). Reductions in herbicide use were between 20 and 40%. In the CBA, we assumed for this PAT a reduction of 27% of herbicide use. The baseline cost of the herbicide is 76 € ha^−1^ and the savings are 0.27 × 76 = 20 € ha^−1^. Soil maps were obtained with a VERIS MSP3 or EM38 soil sensor system. Cost of the map is ca 150 € ha^−1^. The map can be used 3 to 5 years in a row and may have at least 2 uses (in VRA soil herbicides and planting section). We assumed an annual cost of 25 € ha^−1^ in the CBA. Standard practice arable field boom sprayers were used in the VRA field studies, so there were no additional costs for PAT on the machines. PAT for individual section or nozzle control were not studied. An additional benefit of a reduction in soil herbicide use is less growth reduction of the crop, leading to crop yield increases up to 5%. Here, we assumed a yield increase of 3%.

#### Fertilizer Application 2 (N Side Dress)

Soil nitrogen supply varies widely from year to year and from field to field. Potato farmers can respond to the corresponding variability in N-fertilizer requirement by measuring the time course of nitrogen uptake and applying an appropriate amount of side dress N if and when needed. Side dress recommendations based on physical measurements of crop and/or soil are commonly used (van Dijk and van Geel 2012), but the costs of sampling and the time required for analysis of samples are considered limitations of these methods. Also at most, a few samples are taken per field, so the spatial resolution of this method is limited. In contrast, optical measurement of crop N status is quick, cheap and can be done on every square meter of a field thus enabling VRA of N. Results of a crop reflectance-based N-side dress system for potatoes have been published (Booij et al. 2001; van Evert et al. 2012a) showing N use can be reduced by 15% on average without affecting yield or quality.

We made the following assumptions in the CBA. The Yara N-Sensor has been demonstrated to perform well. Other crop reflection light sensors can be used too. We assumed that the N-Sensor use costs are 25 € ha^−1^ year^−1^ (derived from € 25,000 sensor costs, depreciation in 5 years, 100 ha per sensor per year half of its use in other operations (e.g. late blight or haulm killing, and in other crops). Standard N application rate = 250 kg ha^−1^, so saving can be 37.5 kg N ha^−1^, which equals 38 € ha^−1^. We assumed that every kg N that is applied, but not used by the crop, leaches to the groundwater. Thus the reduction in leaching is also 37.5 kg N ha^−1^. Standard practice fertilizer spreaders were used in the VRA field studies, so there are no additional costs for PAT on the machines. PATs on spreaders were not accounted for in the CBA.

#### Crop Protection (Late Blight)

We evaluated in the CBA experimental data from on-farm studies on VRA fungicide use against potato late blight disease (Jongema 2016; Kempenaar et al. 2010, 2016). We did not address other possible VRAs or PATs for detection of other diseases with camera systems, nor decision support systems (DSSs) for control of early blight and other pests and diseases. We only refer to promising phenotyping studies, such as those by Polder et al. (2017).

Late blight in potato is caused by the oomycete *Phytophthora infestans*. To prevent infection and damage, farmers apply fungicides circa every 5 to 10 days in The Netherlands during the season, and a total of 10 to 15 sprays per growing season. Farmers mainly apply fungicides with a preventive mode of action. The timing of fungicide sprays can be based on decision support systems that predict infection periods (Skelsey et al. 2009). Fungicide dose of sprays can be based on the above-ground amount of leaves and stems, as measured with crop reflection sensors. The more biomass present on a part of the field, the higher the dose of the fungicide should be (Kempenaar et al. 2010). VRA fungicide trials in potato showed reductions in herbicide use between 20 and 30%. In the CBA, we assumed an average reduction of 22%. Fungicide products applied were Shirlan, Revus and Infinito. The baseline costs of the fungicides were 383 € ha^−1^ and the savings were 0.22 × 383 = 84 € ha^−1^. We did not assume an effect of the VRA on the yield of the crop. There is an additional cost of obtaining biomass maps and DSSs. We assumed biomass maps were obtained using a Yara N-Sensor or another crop reflection light sensor (Greenseeker, Crop Circle, Fritzmeier) (Kempenaar et al. 2010, 2014b). Costs are given in the N side dress section (25 € ha^−1^ year^−1^). Standard practice arable field boom sprayers were used in the VRA field studies, so there was no additional cost for PATs on the machines. DSS use was considered standard practice in the CBA study region and costs were therefore not accounted for.

#### Irrigation

Although various PATs for precision irrigation are available, such as soil moisture sensors and software for modelling soil moisture balance and irrigation advice, we did not evaluate them because VRA of irrigation water is not a subject in the CBA study region.

#### Potato Haulm Killing

The standard practice method to kill the potato canopy before harvest is application of a defoliant herbicide (Kempenaar and Struik 2008). Herbicide dose can be based on the above-ground amount and activity of the biomass, as measured with crop reflectance light sensors (Kempenaar et al. 2010, 2014b). The more biomass present on a part of the field, the higher the dose of the defoliant should be. VRA potato haulm killing herbicides trials in potatoes showed reductions in herbicide use between 20 and 47%. In the CBA, we assumed an average reduction of 38%. Herbicide products applied were Reglone, Spotlight and Finale. The baseline costs of the herbicides were 68 € ha^−1^ and the savings are 0.38 × 68 = 26 € ha^−1^. We do not assume an effect on the yield of the crop. There are additional costs of obtaining biomass maps and some extra decision support. We assumed in the CBA that this was done using a Yara N-Sensor or other crop reflection light sensor in combination with a standard practice field sprayer. We assumed the costs for applying this VRA at 25 € ha^−1^, as above for VRA of late blight fungicides. Costs of this VRA can be reduced by not with 90% if satellite images are available (Kempenaar et al. 2014b; van Evert et al. 2012b).

#### Selective Harvest and Storage

This PAA is not addressed in the CBA, but may become a useful tool when potato quality sensors become available for use in practice.

### Cost-Benefit Evaluation of Five Emerging PA Applications in Potato

We found experimental data on costs and benefits of VRAs in potato for five crop management actions. Two of them, VRA planting and VRA soil herbicide weed control, used soil maps in combination with other information to arrive at the VR task map. The other three used crop biomass index maps in combination with other information to make the task map. This multiple use of data helps to make the CBA positive, because costs can be spread over several actions and investments will pay off faster. The potato haulm killing VRA was tested in on-farm research most widely. For the others, we have less on-farm data available. It is therefore too early to draw strong conclusions on the costs and benefits of the VRAs. We can conclude that:

- For both the VRA seeding density and VRA soil herbicides, an increase in potato yield is a must to make the CBA positive. Test results showed that yield increases can be expected from these VRAs. The investments pay off when 2–4% yield increase is achieved.
- Costs and savings of VRA potato haulm killing (26 vs 25 € ha^−1^) and N side dress (28 vs 25 € ha^−1^) with biomass maps are in the same order.
- VRA fungicide use in potato has a positive cost-benefit ratio thanks to multiple applications (84 vs 25 € ha^−1^).
- Savings on pesticide and N-fertilizer use were on average about 25% compared to the reference standard practice.

The conclusions are under the assumptions that the costs of sensor data are shared over different VRAs and no extra investments are needed in sprayer and spreader technology. On-farm test results in the CBA were obtained with modern yet standard sprayers and spreaders that can adjust the rate uniformly over the whole width of the spray boom or swath. We see technical developments towards dosing per section or even per nozzle. These technologies require of course higher investments, and will also give higher savings.

We also assumed that the VRA was done with biomass maps of a nearby sensor, as being the first available tool in the market. Since a few years, we can also use satellite and drone data. Costs are in the order of 1 and 20 € ha^−1^, respectively, for individual maps. In some situations, the CBA will be better if remote sensing maps are used instead of nearby sensing maps. If canopy reflectance maps are needed throughout the season, it makes sense to subscribe to a vegetation monitoring system such as Bioscope (ESA funded project).

We foresee a slow but gradual adoption of the five VRA technologies presented in this paper. And more VRAs will become available and adopted. For instance, VRAs of liquid manure, compost and base fertilizer application will be done with soil maps as well when the VRA models are there and validated. We also expect that sensor technology for detection of weeds, diseases and pests will allow curative treatments. This, in combination with the fact that the information and communication technology environment gets better every year, makes that VRAs can have a bright future in potato, not only from an economic point of view but also from an environmental point of view (van Evert et al. submitted).

## Appendix

**Table 2:** Quantitative information on French fries potato production on clay soil in The Netherlands (Anonymous 2015, p. 621)

Category/details	Amount	Unit	Price	Unit	Product/total (€)
Harvested product (a)					*8332*
Potato gross yield	53,560	kg	0.155	€/ha	8332
Variable costs (b)					*2513*
Potato seeds (b1)	2700	kg	0.38	€/ha	756
Fertilizer use (b2)					
Nitrogen	252	kg N	1.05	€/kg N	265
Phosphate	105	kg P_2_O_5_	1.00	€/kg P_2_O_5_	105
Potassium	180	kg K_2_O	0.64	€/kg K_2_O	115
Crop protection (b3)					
Weed control^1^	4.1	kg a.i.^7^			126
Disease control^2^	4.8	kg a.i.			409
Insect control^3^	0.1	kg a.i.			32
Potato haulm killing^4^	0.8	kg a.i.			68
Energy use (b4)					
Fuel (gasoline)	260	l	1.10	€/l	285
Electricity	1071	kWh	0.15	€/kWh	164
Other costs (b5)^5^					188
Margin (a–b)^6^					*5819*
Labour input^7^	30	hours			

^1^Mainly soil herbicides, 1–2 sprays per crop

^2^Late and early blight control, ca 15 sprays per crop

^3^Aphid control, 1–2 sprays per crop

^4^Mainly diquat-dibromide, 1–2 sprays per crop

^5^Including variable costs related to monitoring, storage, sales

^6^Mechanisation, storage facility and labour not yet accounted for

^7^Soil tillage, planting, crop care, harvest and shedding

^8^Prices active ingredients (a.i.) not relevant, totals calculated on pesticide product prices

## Abbreviations

a.i.: Active ingredient
CBA: Cost-benefit assessment
DSS: Decision support system
FMIS: Farm management information system
GIS: Geographical information system
GNSS: Global navigation satellite system
IT: Information technology
NIR: Near-infra-red
PA: Precision agriculture
PAT: Precision agriculture technology
PAA: Precision agriculture application
PF: Precision farming
VR: Variable rate
VRA: Variable rate application
